# Validation of the Greek Version of Euthanasia Attitude Scale (EAS) in Greek Medical Doctors

**DOI:** 10.3390/nursrep12020030

**Published:** 2022-04-16

**Authors:** Maria Malliarou, Vasileios Tzenetidis, Iokasti Papathanasiou, Kiriaki Vourdami, Nikolaos Tzenetidis, Athanasios Nikolentzos, Pavlos Sarafis

**Affiliations:** 1 Laboratory of Education and Research of Trauma Care and Patient Safety, Faculty of Nursing, University of Thessaly, 41110 Larisa, Greece; vtzenetidis@uth.gr (V.T.); iokpapathan@uth.gr (I.P.); 2 Post-Graduate Programme Health Care Management, School of Social Sciences, Hellenic Open University, 26335 Patras, Greece; kvourdami@gmail.com (K.V.); anikolentzos@gmail.com (A.N.); 3 Hellenic Police, 11522 Athens, Greece; tzenranger@gmail.com; 4 General Department of Lamia, University of Thessaly, 35100 Lamia, Greece; psarafis@uth.gr

**Keywords:** euthanasia, instrument validation, physicians, Gr-EAS, Euthanasia Attitude Scale

## Abstract

This study aimed to examine the reliability and validity of the Euthanasia Attitude Scale (EAS) in Greek medical doctors. A cross-sectional study design was conducted, including 120 physicians at clinical setting in 2019 (men 64.5%). A self-report questionnaire, including socio-demographic data and the Euthanasia Attitude Scale, which assesses attitudes towards euthanasia, were used for data collection. The mean (standard deviation) of the EAS were 74.62 (14.33). The Cronbach’s alpha was 0.944 and the confirmatory factor analysis to investigate the validity of the EAS scale, after modification effects, revealed an acceptable adjustment for the questionnaire. The GFI index was above 0.8 and close to 0.9, and the CFI index was above 0.9, which is the acceptable limit. The RMSEA index was acceptable below 0.08. The total Gr-EAS correlated with all five factors (Pearson r = 0.400–0.973, *p* < 0.001). According to the findings of this study, the Euthanasia Attitude Scale is a reliable and valid measure for assessing the attitudes toward euthanasia in Greek physicians. This Greek adaptation will be valuable in future studies examining the attitude of physicians towards euthanasia.

## 1. Introduction

The word euthanasia is derived etymologically from Greek and it means good and peaceful death [1]. The creation of technological achievements that can prolong life, as well as the changes that are observed from generation to generation or intercultural in patients’ views form new moral dilemmas for societies. Patients who “suffer”, fearing that their lives will be prolonged unnecessarily or that they will end up with unbearable discomfort and will probably become a burden to their loved ones [2], express requests to expedite their termination by euthanasia or medical assistance, a fact that provokes conflicting views and reactions within both the scientific community and the general public. The intensity of the problem surrounding euthanasia is reflected in the fact that, in modern bioethics, this issue dominates and claims, together with that of prolonging life, the position of being the most active research field [3]. For those who provide care in terminally ill patients, euthanasia is an open dilemma in every day clinical practice. Even the Hippocratic Oath gives very good reasons both in favor or against euthanasia, when practitioners commit to “do no harm” [4].

Extensive engagement with the issues of euthanasia and physician-assisted suicide has led to the development of numerous definitions of the two concepts that can sometimes be confusing. Among these definitions, the two do not mention anything about the rules and values associated with those defined, nor do they take a position on whether they constitute justified acts of murder by invocation. These are the definitions of the European Association of Palliative Care (EAPC) that have recently been adopted by the International Union for Palliative Care and Palliative Care [5,6]. Euthanasia is the deliberate cessation by a doctor (or other person) of a person’s life of medication, following that person’s voluntary and capable request [7]. Physician-assisted suicide (PAS) is the deliberate assistance by a physician of a person to end his or her life by providing him or her with medicines for self-administration, upon the voluntary and capable request of that person [8,9]. Indicative of the ongoing debate and controversy over euthanasia and medically assisted suicide (MAS) are the growing number of declarations made by various actors in recent years [10].

National and international medical and nursing associations, religious organizations, and political parties have tried, through a total of 62 declarations (45 of which express rejectionist positions), to stimulate public interest and/or call for reforms. At the same time, there are more and more international moves for the legalization of euthanasia and MAS. Voluntary euthanasia (with the patient’s consent) is legal in only few countries, including Canada, New Zealand, Belgium, the Netherlands, Luxemburg, Colombia, Spain, and some States of Australia [11]. In Greece, some forms of euthanasia seem to take place “behind closed doors”, while there is talk of an urgent need to fill legal gaps [12]. In most countries, there are no relevant regulations and only in some legal orders have they become a reality that is protected by the current legislation. Proponents of legalization argue that respect for patient autonomy and therefore the right of critically ill or severely disabled patients to control the time and manner in which they die [13]. Others argue that sometimes it can be the only choice of a person, when, e.g., they want to get rid of incurable suffering, and palliative care or treatment regimens no longer work. According to Verbakel and Jaspers (2010) [14], another reason that euthanasia and MAS are accepted by many (and especially those who have had experience in caring for terminally ill patients) is that they believe that they ensure dignity in the death process. Those who oppose legalization base their arguments on issues of ethical principles and values [15]. Several researchers claim that euthanasia or assisted suicide practices would shake patients’ and society’s confidence in the medical body [16] and would facilitate the end-of-the lives of vulnerable people such as the elderly, the disabled, the mentally ill, and the financially weak, for whom this euthanasia “is not a matter of medical but of social rules, ethics, and law”, as stated in the introductory report of the relevant law passed in the Netherlands. Nevertheless, health professionals hold an important, crucial position and are an integral part of the whole process as they are the ones that patients will usually turn to and who will eventually be called upon to implement these practices. Therefore, studies evaluating their views or experiences and attitudes towards euthanasia would potentially be helpful to the wider dialogue. The EAS scale has been extensively used by researchers. Indicatively, the study of Nortje (2013) [17], who used it in a sample of South Africans of different nationalities, studied the effect that national background has on individuals’ views on euthanasia. Sigh et al. (2015) [18] used it to measure the views of a sample of physicians (oncologists, hematologists, and psychiatrists) from 28 public and private hospitals in Delhi on passive and active euthanasia, while Tang et al. (2010) validated its reliability and validity in a study performed on clinicians in Hong Kong [19]. In addition, Alborzi et al. (2018) used EAS to quantify the views of a sample of ICU nurses so that they could then relate them to the moral discomfort they may experience in the performance of their duties [20]. The aim of this study was to present an adaptation and validation of the Euthanasia Attitude Scale and to evaluate its psychometric properties among a sample of physicians in Greece.

## 2. Materials and Methods

### 2.1. Participants

A convenience sample of 120 physicians in a clinical setting, who were members of the Medical Association of Athens, were invited to participate in the study. Snowball sampling was used to select the study sample (77.5% response rate). A prior sample size calculation was not performed, rather the sample size was based on the entire population of the clinical setting used in our study. The exclusion criteria consisted of those physicians who were not willing to participate in the study. The university’s ethical committee approved the conduct of the study. Completion of the questionnaires lasted 10 min, and no compensation was provided to the participants. Each participant completed a demographics questionnaire regarding age, gender, marital and job status, and years of prior working experience. All participants were reassured that their anonymity and confidentiality would be protected without obtaining any personal, identifying information. They have primarily been informed about all the details of the study (scope, their right to withdraw, being undertaken as part of a master thesis completion).

### 2.2. Instruments

The Euthanasia Attitude Scale (EAS) developed by Holloway, Hayslip, and Murdock [21] is a questionnaire consisting of 30 (16 positive and 14 negatively structured) items related to consent in passive or active euthanasia, the rights of end-stage patients, the place of modern technologies in life preservation, brain death, the role of the physician in the final phase of the patient, and other ethical and legal issues. The answers are given on a four-point Likert scale and can be “strongly agree”, “agree”, “disagree”, or “strongly disagree”. For each participant, the numbers chosen for the 30 sentences are added together creating a score, which can range from 30 to 120. Values <75 are indicative of a negative overall attitude, while values 75–120 reflect positive attitude [18]. The EAS scale is divided to five factors—Factor I “General Orientation towards Euthanasia” (1, 3, 5, 8–10, 16, 20–24, 27, and 28), Factor II “patients’ rights issues” (7, 9, 14, 16, 17, 29, and 30), Factor III “role of life-sustaining technology” (6, 11, 12, 14, and 15), Factor IV “professional’s role” (2, 4, 25, and 26); Factor V “ethics and values” (1, 3, 10, 18, 19)—with excellent psychometric properties, possessing stability over time, internal consistency, and discriminant validity [21].

### 2.3. Procedure

Permission to translate the original English version of the Euthanasia Attitude Scale into Greek for research purposes was obtained from one of the authors (Bert Hayslip). The translation was done using the guidelines for the process of cross-cultural adaptation of self-report measures [22]. The EAS was initially translated into Greek independently by two physicians, who were fluent in English. The two drafts were then compared item by item until the consensus was reached through discussion. The revised draft of the Greek version of the EAS (Gr-EAS) that was agreed upon was then translated back into English by two bilingual individuals who had no prior knowledge of the instrument. The back translations were compared, and inconsistencies were addressed until a final back-translation document was agreed upon. This version was then compared with the original English version of the EAS for final confirmation of the linguistic accuracy. The backward translation was also checked after a pilot test of the questionnaire in Greek in a sample of 20 people, in order to capture problems regarding the wording and comprehension of the questions (if they did not understand a word, if a word or expression was offensive or unacceptable, etc.). This resulted in the final version of the Greek EAS questionnaire.

### 2.4. Data Analysis

Descriptive statistics were used to describe the characteristics of the sample, such as mean, standard deviation (SD), maximum (max), and minimum value (min). The Pearson’s r test was also performed. The Pearson correlation coefficient was used to measure the strength of a linear association between two variables, where an value r = 1 means a perfect positive correlation and a value of r = −1 means a perfect negative correlation. Cronbach’s alpha coefficient was used to examine the internal consistency of each of the subscales with the customary level of >0.070, reflecting a satisfactory internal consistency [23]. Finally, confirmatory factor analysis was used to check the validity of the EAS scale with a maximum likelihood procedure, in which the CFI (comparative fit index), GFI (goodness of fit index), and RMSEA (root mean) were evaluated (square error of approximation) [24]. CFI and GFI indices can have values from 0 to 1, and it is considered that there is a good fit to the data when it is close to or above 0.9, or with even stricter criteria when it is close to or above 0.95 [25]. The CFI index is considered more appropriate for model estimation as it takes into account the sample size. In addition, for the GFI index, a limit of 0.8 has been proposed for good adjustment. RMSEA values less than 0.05 indicate a good adjustment and values up to 0.08 indicate an acceptable adjustment. Data analysis was performed using the Statistical Package for Social Sciences (SPSS) (version 22, Armonk, NY, USA). The level of statistical significance was set at 5%.

## 3. Results

Here, 93 physicians took part in the present study, with a mean age of 53.7 years (SD = 7.4). Most were men (64.5%) with 25.4 (SD = 8.0) years of service on average. While the vast majority of the sample (95.7%) embraced the Orthodox religion, three doctors (3.25%) declared themselves atheists and one (1.1%) stated something else. Of the sample, 39.8% stated that they come in contact with an end-stage patient two to three times a year, while 25.8% stated this occurred once every 2–3 months. About 53% of participants cared for 1–10 dying patients in the last 12 months, while 33.3% did not care for a dying patient. Years of service, frequency, and number of dying patients treated were independently associated with EAS score, while gender and age were not (Table 1). Religion is one of the most studied variables that influence attitudes towards euthanasia, but in our sample, all physicians embraced the Orthodox religion, while only four of them considered themselves as atheists. Only three of them reported that they had some kind of training in treating end-of-life patients. Physicians who needed training on psychological support in end-of-life patients scored higher in EAS total score.

Means of individual items of Gr-EAS ranged from 2.02 to 3.39. (Table 2). In Table 2 standard deviations, item homogeneity and a if item is deleted are also described. Homogeneity index was found between 0.001 and 0.826. Cronbach α values for EAS, if item deleted, ranged from 0.939 to 0.949.

### 3.1. Confirmatory Factor Analysis

The Shapiro–Wilk test for normality was statistically significant (*p* = 0.000) for all items. An examination of the Kaiser−Meyer Olkin measure of sampling adequacy suggested that the sample was factorable (KMO = 0.868). The Bartlett’s test of sphericity was significant (*p* = 0.000). The confirmatory factor analysis performed to investigate the validity of the EAS scale, after modification effects, revealed an acceptable adjustment for the questionnaire, where the GFI index was above 0.8 and close to 0.9 and the CFI index was above 0.9, which is the acceptable limit. The RMSEA index was acceptable below 0.08. Descriptive statistics of the Gr-EAS questionnaire and its five factors are given below (Table 3). The mean value of the Gr-EAS total was found to be 74.62 (SD 14.33).

The intercorrelation matrix for the Gr-EAS dimensions is presented below (Table 4). Of note is the strong positive association between general orientation towards euthanasia, patients’ rights issues, role of life sustaining technology, and ethics and values, accordingly with attitude towards euthanasia (Gr-EAS total).

In order to calculate reliability of Gr-EAS, we used the method of internal consistency. The resulting reliability coefficient was very high (Cronbach’s alpha = 0.944), which indicates the consistency and stability of the measurement and indicates that all items measured the same construct.

### 3.2. Internal Consistency Reliability

Cronbach’s α for Gr-EAS total was found to be 0.944, while each of the five factors of the scale was above 0.7 (Table 5), which shows that there was an acceptable reliability of the questionnaire

## 4. Discussion

Holloway primarily developed the Euthanasia Attitudes Scale in order to measure and assess attitudes toward both passive and active euthanasia, significant in both an educational and a didactic sense to the extent that individuals not only rarely confront their feelings about and attitudes toward euthanasia, but also seldom take the opportunity to share such feelings with significant others [21]. The scale developed was also designed to reflect the complexity of situations in which euthanasia decisions might be made. Holloway recognized the complexity of the issue and created a 30-item questionnaire which included many issues involved in euthanasia, such as views about the professional’s role, patients’ rights, and active versus passive euthanasia [21]. They concluded in their study that, although persons often express fairly strong attitudes toward euthanasia, they are not fixed and indeed can change significantly via death education within a fairly brief period of time. In this study, we translated the Euthanasia Attitude Scale (EAS) and then assessed its internal reliability and construct validity, performing confirmatory factor analysis to create a valid Greek version of the survey. Validation of this questionnaire will be used for further studies as there are no existing tools for measuring the attitudes towards euthanasia in Greece. Physicians’ attitudes towards euthanasia are well described, in that they do not lie only in their values, but also their education, and will have an impact on how they are going to deal with possible medical dilemmas. Τhe the Greek version of Euthanasia Attitude Scale (EAS), has good internal consistency, and the Cronbach’s alpha ranged from 0.710 to 0.951 for the five factors, while the total Gr-EAS was found to be 0.944. The minimum required was 0.7. The confirmatory factor analysis performed to investigate the validity of the EAS scale, after modification effects, revealed an acceptable adjustment for the questionnaire, where the GFI index was above 0.8 and close to 0.9, and the CFI index was above 0.9, which is the acceptable limit. The RMSEA index was acceptable below 0.08. The EAS total score was sensitive to years of service, frequency and number of dying patients treated, and need for training on psychological support in end-of-life patients, but not to age and gender. Onieva-Zafra also stated that beliefs of euthanasia can change and vary according to individual education and experience [26]. The same study that aimed to evaluate EAS scale psychometric properties among a sample of nursing students in Spain performed an exploratory factor analysis and found that the Kaiser–Meyer–Olkin index of sampling adequacy was 0.905 and the Bartlett’s Test of Sphericity was 2972.79 (*p* < 0.001). The factorial solution comprised four domains and the scale demonstrated adequate internal consistency (Cronbach’s alpha ¼ 0.878). The initial structure based on four domains was conserved, with a factorial solution that explained 52.79% of the total variance [26]. However, another study that aimed to examine the reliability and validity of the Euthanasia Attitude Scale (EAS) in Hong Kong medical doctors found a higher percentage of total variance (62.10%) [19].

## 5. Conclusions

According to the findings of this study, the Euthanasia Attitude Scale is a reliable and valid measure for assessing attitudes toward euthanasia in Greek physicians. Despite its limitations, Gr-EAS is recommended for use because of its good psychometric properties. Τhe Gr-EAS questionnaire will be valuable in future studies examining the attitude of physicians towards euthanasia.

## Figures and Tables

**Table 1:** Associations with the total score of the Euthanasia Attitude Scale (EAS).

Category	*N*	Mean	SD	*p*-Value
Gender				
Male	60	74.68	13.88	0.201
Female	33	74.52	15.32	
Years of service				
≤20	28	79.93	13.37	0.037
21–30	43	73.60	14.37	
>30	22	69.86	13.89	
Age				
≤50	31	78.68	13.69	0.156
51–60	43	72.63	13.09	
61+	19	72.53	17.14	
Treating dying patients				
2–3 times a year	48	77.17	15.01	0.025
Once every 2–3 months	24	75.96	13.57	
More than once a month	21	67.29	11.31	
Number or dying patients				
0	31	77.74	16.711	0.016
1–10	49	75.35	12.321	
11+	13	64.46	11.377	
Need for training on psychological support in end-of-life patients				
no	37	73.70	12.558	0.060
yes	56	75.23	15.467	

**Table 2:** Means (M) and standard deviations (SD), item homogeneity, if an item is deleted, skewness, and kurtosis of EAS items.

Items	Mean	SD	Item−Total Correlation	Cronbach’s Alpha if Item Deleted	Skewness	Kurtosis
1. Even if death is positively preferable to life in the judgment of a terminal patient, no action should be taken to induce the patient’s death.	2.02	0.847	0.717	0.941	0.508	−0.314
2. Under any circumstances I believe that physicians should try to prolong the lives of their patients	2.60	0.694	0.157	0.949	0.323	−0.393
3. To me there is absolutely no justification for ending the lives of persons, even though they are terminally ill.	2.16	0.798	0.721	0.941	0.224	−0.434
4. Some patients receive “comfort measures only” (for example. pain relieving drugs) and are allowed to die in peace without further life extending treatment. This practice should be prohibited.	2.81	0.664	0.107	0.946	−0.905	1.466
5. I believe it is more humane to take the life of an individual who is terminally ill and in severe pain than to allow him/her to suffer.	2.25	0.702	0.652	0.942	0.385	0.256
6. An individual who is “brain dead” should be kept alive with proper medical intervention.	2.95	0.728	0.547	0.943	−0.436	0.245
7. I believe that a person with a terminal and painful disease should have the right to refuse life-sustaining treatments.	2.81	0.557	0.458	0.943	−0.049	−0.098
8. I bear no ill feelings toward a person who hastens the death of a loved one to spare the loved one further unbearable physical pain.	2.24	0.902	0.751	0.940	0.238	−0.717
9. I believe there should be legal avenues by which an individual could pre-authorize their own death in case intolerable illnesses arises.	2.65	0.816	0.697	0.941	0.008	−0.544
10. I cannot envision any medical circumstance in which the termination of life would be merciful.	2.37	0.857	0.615	0.942	−0.047	−0.691
11. I would support the decision to reject additional treatments if a dying person contracts a secondary disease that is sure to bring about a quick and painless death.	2.58	0.665	0.380	0.944	0.491	−0.424
12. I would support a doctor’s decision to reject extraordinary measures if a patient has no chance of survival.	2.42	0.681	0.342	0.944	0.299	−0.029
13. I support the decision to provide “comfort measures only” if a terminally ill patient is dying and has only a few hours of life left.	3.39	0.590	0.170	0.946	−0.358	−0.679
14. If I were faced with the prospect of having a loved one suffer a slow and painful death, I would support his/her decision to refuse further medical life-sustaining treatment.	2.73	0.782	0.608	0.942	0.097	−0.663
15. To me it is an act of mercy to a living but “brain dead” person to turn off life-sustaining machines.	2.54	0.841	0.649	0.942	−0.177	−0.514
16. If I were faced with the situation of suffering a slow and painful death, I should have the right to choose to end my life in the fastest and easiest way possible.	2.63	0.844	0.792	0.940	0.119	−0.698
17. It is cruel to prolong intense suffering for someone who is mortally ill and desires to die	2.83	0.717	0.504	0.943	−0.276	0.033
18. No one, including medical professionals, should be allowed to decide to end a suffering person’s life.	1.77	0.898	0.798	0.940	0.649	−0.993
19. To me, anyone who assists a suffering and terminally ill person to die is nothing but a common murderer.	2.51	0.892	0.718	0.941	0.030	−0.707
20. A terminally ill person who is in severe pain deserves the right to have his/her life ended in the easiest way possible.	2.48	0.731	0.593	0.942	0.483	−0.189
21. If a friend of mine were in severe pain, close to death, and begged me to try to convince the doctors to end his/her life mercifully I would ignore their plea.	2.10	0.945	0.752	0.941	0.119	−1.312
22. The injection of a lethal dose of some drug to a person in order to prevent that person from dying an unbearably painful death is unethical.	1.89	0.926	0.823	0.939	0.553	−0.901
23. No matter how much a person might plead for death to avoid unbearable pain, no one should assist the person to accomplish his/her wish.	2.03	0.938	0.826	0.939	0.420	−0.873
24. Inducing death for merciful reasons is acceptable.	2.11	0.758	0.785	0.940	0.275	−0.237
25. Terminally ill patients who try to starve themselves to death to avoid unbearable pain should be forcefully fed intravenously.	3.18	0.658	0.001	0.947	−0.445	0.310
26. For me, it is unethical to allow the termination of a human life when medical technology is able to preserve it.	2.34	0.617	0.609	0.942	0.478	0.264
27. The termination of a person’s life, done as an act of mercy, is unacceptable to me.	2.05	0.889	0.820	0.940	0.178	−1.145
28. Assisting a person who faces a future life of unbearable pain to end his/her life is murder, as I see it.	2.33	0.771	0.750	0.941	0.216	−0.223
29. One should have the right to choose to die if he/she is terminally ill and is suffering.	2.73	0.739	0.668	0.941	−0.183	−0.165
30. A terminally ill individual should be allowed to reject life support systems.	3.11	0.521	0.336	0.944	−0.327	2.914

**Table 3:** Descriptive statistics of the Gr-EAS and its factors.

	Mean	SD	Min	Max
General orientation towards euthanasia	24.1	7.4	11	42
Patients’ rights issues	19.5	3.5	12	28
Role of life sustaining technology	13.2	2.5	7	19
Professional’s role	10.9	1.1	8	14
Ethics and values	11.5	3.2	5	19
Gr-EAS total	74.62	14.33	55	109

**Table 4:** Intercorrelations between the Gr-EAS factors and Gr-EAS total.

		Patients’ Rights Issues	Role of Life Sustaining Technology	Professional’s Role	Ethics and Values	Gr-EASTotal
General orientation towards Euthanasia	r	0.841	0.723	0.311	0.926	0.973
	*p*	0.000	0.000	0.002	0.000	0.000
Patients’ rights issues	r		0.770	0.262	0.783	0.897
	*p*		0.000	0.011	0.000	0.000
Role of life sustaining technology	r			0.293	0.695	0.828
	*p*			0.004	0.000	0.000
Professional’s role	r				0.346	0.400
	*p*				0.001	0.000
Ethics and values	r					0.933
	*p*					0.000

**Table 5:** Cronbach’s α for Gr-EAS and the five factors.

	Cronbach’s α
General orientation towards Euthanasia (14 items)	0.951
Patients’ rights issues (7 items)	0.823
Role of life sustaining technology (5 items)	0.710
Professional’s role (4 items)	0.710
Ethics and values (5 items)	0.855
Gr-EAS total	0.944
